# A Measurement Invariance Investigation of the Polish Version of the Dual Filial-Piety Scale (DFPS-PL): Student-Employee and Gender Differences in Filial Beliefs

**DOI:** 10.3389/fpsyg.2021.713395

**Published:** 2021-09-28

**Authors:** Joanna Różycka-Tran, Paweł Jurek, Michał Olech, Tadeusz Dmochowski

**Affiliations:** ^1^Institute of Psychology, University of Gdansk, Gdańsk, Poland; ^2^Department of Psychology, Medical University in Gdansk, Gdańsk, Poland; ^3^Institute of Political Sciences, University of Gdansk, Gdańsk, Poland

**Keywords:** filial piety, DFPS-PL, measurement invariance, cultural psychology, gender, employee

## Abstract

Filial beliefs are defined as a cognitive script or even a contextualized personality construct for social exchanges, which shapes the attitudes of individuals. In the given study, we investigate the factorial structure of the Polish version of the Dual Filial Piety Scale (DFPS-PL) and verify whether measurement of filial piety is invariant among students and employees, and among men and women. Two studies were conducted on different age samples: 489 students aged 18–24 and 849 employees aged 25–64. In order to verify the hypotheses, the DFPS-PL was administered. As a result of confirmatory factor analyses (CFA), it has been demonstrated that the structure of filial piety measured by the DFPS-PL among students and employees, and men and women, could be interpreted as two-factorial, and that there is partial scalar measurement invariance for the tested model across these groups (MGCFA). The comparison of the average latent mean scores suggests that employees declare a lower level of AFP (Authoritarian Filial Piety; need of social belonging and collective identity) than students. There were no significant differences between students and employees when RFP (Reciprocal Filial Piety; need of interpersonal relatedness) was compared. In addition, the results showed that women score higher in RFP than men. The given findings are discussed in the context of values transition in non-Asian countries. The main contribution is to confirm the factorial structure of the DFPS-PL and introduce the novel Eastern concept of Filial Piety to Western culture.

## Introduction

Traditional views of filial beliefs (*filial piety*) refer to the attitudes of children toward how they should treat their parents, as well as an emphasis on respect and care for elders, containing important ideas about social relations (Ho, 1986; Yeh, 2003). In modern psychological studies, we can observe the evolution of the conceptualization of filial piety: initially treated as a Chinese value-based cultural norm; nowadays, filial piety is viewed as universal construct, defined as a cognitive script or even a contextualized personality construct for social exchanges (Bedford and Yeh, 2019, 2021). Having considered the foregoing, the concept of filial piety was transferred from the cultural dimension (*emic*) to the personality dimension (*etic*), demonstrating the universal mechanism of child-parent relationships found in diverse cultures, such as Korea, Japan, Malaysia, Thailand, Vietnam, USA, and even Poland.

In contemporary studies, the most popular model is the Dual Filial Piety Model (DFPM; Yeh, 2003), composed of two higher-order factors that correspond to the two focal filial piety attributes in the parent-child interaction: horizontal reciprocal filial piety (RFP), i.e., need of interpersonal relatedness, and vertical authoritarian filial piety (AFP), i.e., need of social belonging and collective identity, which have been shown to have distinct implications for social adaptation and psychological functioning of individuals (see: Yeh and Bedford, 2004; Yeh, 2017; Truong et al., 2020, for review). This model “links the surface content of a cultural norm at the collective level to its underlying psychological needs at the individual level” (Tsao and Yeh, 2019, p. 197). In other words, the psychological function of filial piety is linked to personal motives of children to care for their parents (a universal mechanism), but depending on the cultural context—which reinforces rights and well-being of parents to varying degrees (cultural specificity). Reconceptualizing filial piety has had numerous benefits: It reveals the vertical-horizontal duality of parent-child relationship and at the same time, highlights individual differences in patterns of interaction with parents—as a specific personality trait that is recorded as a response to kin relationships early in the life of a child (the social relationship matrix), also enables research in the field of cross-cultural psychology.

Modern studies confirmed that filial beliefs provide the social and ethical foundations for maintaining social order and influence: moral decision-making (Yeh and Bedford, 2020), academic choices (Hui et al., 2011), motivation and academic achievement (Chen and Wong, 2014; Różycka-Tran et al., 2021; Sappor, 2021), psychosocial adjustment (Leung et al., 2010), or leadership and organizational culture (Low and Ang, 2012). What is more, filial beliefs are correlated (RFP negatively/AFP positively) with hostile attribution bias and cyberbullying perpetration (Wei and Liu, 2020); related with lower psychological difficulties, behavioral problems, and hyperactivity(Ismail et al., 2009); RFP is positively associated with life satisfaction and social competence, while AFP is negatively associated with the self-esteem and social competence of children (Leung et al., 2010; Sun et al., 2019); RFP and AFP positively moderate the relationship between work stress and turnover intention to leave the employer through job satisfaction (Li et al., 2021).

### Filial Piety Among Employees and Students

Filial piety is an important psychological construct because young adults play a number of social roles (e.g., an employee or a leader). According to career construction theory (Savickas, 1995), cultural beliefs represent a social construct that may shape the career path of an individual in the future. It is believed that social environment, including the family of an individual, neighborhood, and school, as well as cultural norms, interacts and influences the career of the individual (Savickas, 2011); it was found that filial piety of employees was positively related to their task performance and organizational citizenship behavior or that career-related RFP (but not AFP) was regarded as important and was associated with career adaptability dimensions (Porfeli and Savickas, 2012). However, because the filial concept comes from Asian culture with a patriarchal system (Hu and Chou, 2000; Liu and Kendig, 2000), it seems that the gender differences in caregiving probably reflect the patriarchal values, where women as wives are expected to perform caregiving duties for their husbands, as sons of aging parents and relatives (Chappell and Kusch, 2007) and could be different in more egalitarian cultures.

Based on these findings, we investigated the filial piety beliefs among employees (regardless of gender) and students. In Poland, parents generally expect obedience from their children but ultimately want them to be independent and self-reliant (Evason, 2017). As the children continue their education, living at home and are dependent on their parents, they are expected to obey the orders and act according to the wishes of their parents. After moving away from home, starting a professional career and starting a family, expectations toward children change dramatically. Parents expect to maintain interpersonal relatedness, but they do not interfere so much in the lives of adult children. In Polish society, the turning point in the life of an adult, affecting the relationship with parents, is starting living with a partner (Żadkowska et al., 2018) and getting married, which for those who continue education, generally occurs after graduation. Graduation is also the turning point of students entering society; it is associated with the start of a permanent job in the learned profession. For adult-working Polish, work very often becomes one of the central values in life (Grabowski, 2016), which redefines their responsibilities and social roles. On the one hand, being employed consumes time and efforts; on the other, it gives a sense of autonomy and independence, including from parents (Greenhaus and Beutell, 1985). Hence, we hypothesize that employees declare lower AFP than do students, and both groups will not differ in terms of RFP since these beliefs are also beneficial from the perspective of working life (see Porfeli and Savickas, 2012).

### Filial Piety and Gender

Because Western culture is more egalitarian (less patriarchal) and is especially fluid with respect to stereotyping and gender roles in society (World Economic Forum, 2020), we investigated the differences between Polish men and women in filial beliefs as they stand today, especially in the work place. Although previous studies had pointed out that adult daughters provide more assistance for their elderly parents than do adult sons (i.e., Zhan and Montgomery, 2003), this finding did not mean that the women have a higher level of filial piety than do the men. In Poland, women describe themselves as more communal than men (e.g., Kosakowska-Berezecka, 2012), which is associated with their greater identification with role, including caring, supporting, and integrating the family. Although the caregiving practices could be urged by both RFP and AFP beliefs, only RFP (as driven by the relational need for social connections) is consistent with stereotypical expectations toward Polish women of being warm, empathetic, and forgiving. Hence, we hypothesize that Polish men declare lower RFP than women. However, we do not expect gender differences regarding AFP since, in the Polish context (a moderately gender equality country, see World Economic Forum, 2020), women and men are equally likely to endorse/not endorse authoritarian values (see Brandt and Henry, 2012).

In our opinion, already-described studies on the literature of filial beliefs (coming from different samples) suggest that the filial piety construct is rather a universal construct; however, it is underlined by specific psychological motivations that depend on culture, in a fashion similar to values (see: Różycka-Tran et al., 2017). So, in the given study, we would like to introduce the concept that filial piety beliefs are represented also by Polish people (universality) but are shaped by specific factors (e.g., employment or gender). In order to perform tests of mean differences, the invariance of the Polish version of the Dual Filial Piety Scale (DFPS-PL) must be evaluated across different social groups, so we investigated if filial piety could be explained by two dimensions (i.e., reciprocal and authoritarian) in the Polish culture and if DFPS-PL demonstrates scalar measurement invariance across student-employee and gender groups.

### Measures of Filial Piety

Since the beginning of the psychological research on filial piety in the 1970s, several measures have been employed in past studies to evaluate filial piety, and many standardized tools for measuring filial piety have emerged (e.g., Ho and Lee, 1974; Yang et al., 1989; Sung, 1995) that underline different aspects of this construct, e.g.: the Filial Behavior Scale (Chen et al., 2007), the Filial Expectation Scale (Wang et al., 2010), or the Filial Piety Scale for Chinese Elders (FPSCE; Fu et al., 2020).

For example, Ho (1994) developed the Filial Piety Scale, which focuses on attitudes toward filial piety, but it overlooks the actual filial behaviors of individuals, or the aspect of love and gratitude that benefit adaptation of adolescents. Gallois et al. (1999) developed a filial piety questionnaire, which emphasizes the cognitive aspect related to subjective norms. However, the questionnaire paid little attention to the underlying motives or reasons for filial behaviors and focuses more on an authoritarian component. To address the limitations of the abovementioned scales and to resolve the beneficial and harmful effects of the filial piety debate (see: Yeh, 2003), Yeh and Bedford (2003) developed the Dual Filial Piety Scale (DFPS), consisting of 16 items loaded on two subscales to measure reciprocal and authoritarian filial piety based on earlier studies (Yang et al., 1989; Yeh, 1997), which was found to have a good model fit in different samples (e.g., Yeh et al., 2013).

Because of cultural change, some authors claim that a majority of people no longer regard filial piety as an authoritative obligation in the twenty-first century. Although newer measures, such as the Contemporary Filial Piety Scale (CFPS, Lum et al., 2015) and the Three-Dimensional Filial Piety Model (TDFPM, Shi and Wang, 2019) are available, there are limitations to these measures, which hinder their applicability across cultures. It seems that “DFPM has been the most important theory, and the DFPS has been the most widely used scale in current filial piety research thus far” (Shi and Wang, 2019, p. 2). This is the reason why DFPS is translated to many languages, e.g., Chinese (Fu et al., 2020), Malay (FPS-M; Tan et al., 2019), South Korean (Sung, 1995), Spanish (Kao and Travis, 2005), Arabic (AFPS; Khalaila, 2010), Vietnamese (DFPS-V; Truong et al., 2020), and now, Polish (DFPS-PL). The factorial structure of the DFPS-PL as an example of the Western culture was not investigated before. What is more, so far, there has been no study on the investigation of group differences of filial beliefs in society (e.g., student-employee or between gender groups).

## Current Study

This article aimed to investigate the invariance and difference of the DFPS-PL between Polish students and employees as well as those between men and women. These two groups seem perfect as a “vehicle” for filial piety values, as parent-child relationships translate into later hierarchical employer vs. employee relationships, where there may be differences based on occupational position or gender. Poland is a country aspiring to egalitarian gender equality, especially in the professional sphere; therefore, it was interesting to study the level of filial piety separately among men and women. Additionally, in order to perform tests of mean differences, the invariance of the DFPS-PL must be evaluated, and latent mean differences must be investigated across different social groups in this scale.

The current paper aims to: (1) investigate the factorial structure of the DFPS-PL; (2) verify whether measurement of filial piety is invariant among students and employees, as well as among men and women; and (3) test the student-employee and gender differences in filial beliefs in the Western culture. Referring to the foregoing research purposes, we hypothesize that the two-factor filial piety model in the Polish population fits substantially better than the one-factor solution. Our hypotheses are based on the DFPM developed by Yeh and Bedford (2003), who found that two distinctive factors, fundamental values underlying the filial piety concept (RFP, AFP), are not mutually exclusive but coexist within an individual and may promote the same outcome (Bedford and Yeh, 2019).

We hypothesize that measurement of filial piety is invariant in the samples of students and in employees, and the samples of male and female, as well, i.e., we expect that the meaning and understanding of reciprocal and authoritarian filial piety should be similar in the above-mentioned samples, since filial piety is considered as a strong belief that is shaped by the culture in which an individual grows up (Bedford and Yeh, 2019). We hypothesize also that employees declare lower AFP but not lower RFP than do students, and men declare lower RFP but not lower AFP than women (see rationale in the *Introduction* section).

## Method

### Participants and Procedure

The back-to-back translation procedure was utilized to develop the Polish version of the DFPS-PL and followed the recommendations of ITC Guidelines for Translating and Adapting Tests (International Test Commission, 2017). Specifically, the original English version was first translated into Polish by a bilingual psychology lecturer. Every effort was made to ensure semantic, idiomatic, and conceptual equivalence, and to preserve overall meaning and nuance. Next, the translated Polish version was then back-translated into English without referring to the original English version by another bilingual language expert. Then, both original and translated versions of DFPS were compared to ensure that the items were consistent. The authorisation of the translation of the DFPS was approved by the author, Kuang-Hui Yeh.

In the current study, a mix of non-probability sampling techniques was incorporated. A group of research assistants was asked to send a survey invitation to people who met one of the following criteria: being employed for at least 1 year, or being a full-time undergraduate or graduate student.

#### Sample of Students

We collected data from 489 students (356 females and 133 males) aged from 18 to 24 years (*M* = 20.38, *SD* = 1.27) from various universities all over Poland, who completed an on-line or paper-pencil version of the scale. The questionnaire was preceded by demographic information and instructions for everyone.

#### Sample of Employees

We collected data from 849 employees (580 females and 269 males) from a variety of industries operating in various regions of Poland. Some of these organisations are global companies with branches on the Polish market. Participants aged from 25 to 64 years (*M* = 37.06, *SD* = 10.08) completed an on-line or paper-pencil version of the scale supplemented with demographic questions. The sample included 132 managers, 426 specialists, and 291 employees in entry-level positions. As it can be seen, the employee sample is highly heterogeneous in terms of age, which may indicate the variety of life stages of the respondents. Considering the developmental periods were identified by Levinson (1978), the participants could be divided into the following age groups: early adulthood, age 25–39 (*n* = 516, 61%); middle adulthood, age 40–59 (*n* = 313, 37%), and late adulthood, age 60 onwards (*n* = 20, 2%).

The protocol of this study was approved by the Ethics Board for Research Projects at the Institute of Psychology, University of Gdansk. According to the local law of different universities, no written permission from the participants was required, as data were collected and analysed anonymously. The participants were assured that their data would remain anonymous and confidential, as we followed APA standards and the Declaration of Helsinki during data collection.

### Measures

The participants filled in the DFPS-PL, which consists of 16 items developed by Yeh and Bedford (2003). Eight items measure reciprocal and another eight items authoritarian filial piety. The respondents indicated how important each statement was to them, using seven-point Likert scale ranging from 1 (strongly disagree) to 7 (strongly agree). Examples of the items measuring reciprocal filial piety include “Be grateful to parents for raising you”; the authoritarian items include” “Live with parents even after marriage” (for all scale items and their translation, see **Table 2**).

### Statistical Analyses

We used R environment (R Core Team, 2020) and lavaan package (Rosseel, 2012) to conduct confirmatory factor analysis (CFA) using maximum likelihood estimation with robust standard errors and a Satorra-Bentler scaled test statistic (MLM), which is better suited for non-normality of the data. First, two models were tested for a total sample, including a two-factor model, which was proposed by the authors of the original scale, and a one-factor model. Overall, the model fit was evaluated using the comparative fit index (CFI), root mean square error of approximation (RMSEA), and standardised root mean square residual (SRMR). Although there are no universally accepted metrics of the model fit (McDonald, 2010), higher values indicate a better fit for the CFI, whereas lower values indicate a better fit for the RMSEA and SRMR. The following criteria for an adequate model fit were adopted: CFI > 0.90 and RMSEA and SRMR <0.08 (Kline, 2016). Model-based reliability was estimated with coefficient omega (McDonald, 1999).

Second, we assessed through multigroup confirmatory factor analysis (MGCFA) three levels of measurement invariance across the compared samples: the configural invariance requires that a given set of indicators is predicted by the same latent variables with the same pattern of factor loadings; metric invariance requires that factor loadings are equal across the groups; and scalar invariance requires that factor loadings and all intercepts are equal across the groups (e.g., Milfont and Fisher, 2010). Partial invariance is established when the parameters of at least two indicators per construct are equal across groups (Byrne et al., 1989). We started investigating measurement invariance by testing for configural invariance across samples, using commonly used criteria to assess goodness of fit of the models. Next, to identify metric and scalar measurement invariance, we used the cut-off criteria suggested by Chen (2007): ΔCFI < 0.01 and ΔRMSEA < 0.015.

Finally, in order to test student-employee and gender differences in filial piety, we conducted a comparison in standardised latent mean scores in groups.

## Results

### Structural Validity of the Polish Version of the Dual Filial Piety Scale

Table 1 presents descriptive statistics for each item of the DFPS-PL in the total sample.

**Table 1:** Descriptive statistics for DFPS-PL items.

	**Item**	**FP factor**	**M**	**SD**	**Skewness**	**Kurtosis**
1	Be frequently concerned about my parent's health conditions/ Interesowaniesiȩzdrowiemikondycja̧rodziców	RFP	6.15	1.19	−1.81	3.85
2	Take my parents' suggestionsevenwhen I do not agree with them/ Uwzglȩdnianie opinii rodziców, nawet jeżeli siȩ z nimi nie zgadzam	AFP	4.62	1.61	−0.55	−0.28
3	Talk frequently with my parents to understand their thoughts and feelings/ Czȩsterozmawianie z rodzicami aby lepiej ich rozumieć	RFP	5.01	1.60	−0.66	−0.16
4	Let my income be handled by my parentsbeforemarriage/ Pozwalanie rodzicom by kontrolowali moje dochody przed małżeństwem	AFP	2.11	1.52	1.37	1.11
5	Be frequently concerned about my parents' general well-being/ Interesowaniesiȩsamopoczuciemrodziców	RFP	5.87	1.36	−1.54	2.51
6	Disregardpromises to friends in order to obey my parents/ Stawianie posłuszeństwa rodzicom ponad lojalność wobec przyjaciół	AFP	3.16	1.62	0.29	−0.67
7	Be concerned about my parents as well as understand them/ Okazywanietroskiizrozumieniarodzicom	RFP	5.72	1.37	−1.37	2.03
8	Give up my aspirations to meet my parents' expectations/ Rezygnacja z własnych aspiracji, aby spełnić oczekiwania rodziców	AFP	2.41	1.59	0.98	0.10
9	Support my parents' livelihood to make their life more comfortable/ Wspieranie rodziców aby żyli bardziej komfortowo	RFP	5.33	1.52	−0.95	0.51
10	Do whatever my parentsaskrightaway/ Natychmiastowe wykonywanie poleceń rodziców	AFP	2.98	1.59	0.38	−0.75
11	Be grateful to my parents for raising me/ Okazywanie wdziȩczności wobec rodziców za to, że mnie wychowali	RFP	5.29	1.65	−1.00	0.38
12	Avoidgettingmarried to someone my parentsdislike/ Unikanie małżeństwa z partnerem/partnerka̧ którego/której rodzice nie lubia̧	AFP	2.15	1.48	1.23	0.82
13	Hurryhome upon the death of my parents, regardless of how far away I am/ Bezzwłoczne pojawienie siȩ w domu rodzica po jego śmierci, niezależnie jak daleko jestem	RFP	6.09	1.57	−1.85	2.64
14	Have at least one son for the succession of the family name/ Posiadaniesyna w celuzachowanianazwiskarodowego	AFP	2.26	1.83	1.32	0.51
15	Take the initiative to assist my parents when they are busy/ Pomaganie z własnej inicjatywy rodzicom w ich obowia̧zkach	RFP	5.25	1.55	−0.92	0.45
16	Live with my parents (or parents-in-low) when married/ Mieszkanie z rodzicami po zawarciu małżeństwa	AFP	1.69	1.33	2.20	4.46

*N, 1,338; RFP, reciprocal filial piety; AFP, authoritarian filial piety*.

Although we found that the two-factor model fit substantially better than the one-factor (see Table 2), it does not meet the adopted fit criteria. Using the “modification Indices” function, we determined that item 2 significantly loads both AFP and RFP factors, so we decided to skip this item in the next models. As can be seen in Table 2, considering the 15-item version of the scale, the two-factor model fit substantially better than the one-factor.

**Table 2:** CFA fit statistics for the two structural models of the DFPS-PL (total sample).

**No of items**	**Model**	**Chi-square**	***df***	**CFI**	**SRMR**	**RMSEA**	**RMSEA 90% CI**
16	One-factor	2,116.79	104	0.734	0.127	0.135	0.130–0.140
	Two-factor	894.04	103	0.898	0.086	0.084	0.079–0.089
15 (without item 2)	One-factor	1,870.87	90	0.741	0.132	0.136	0.131–0.142
	Two-factor	456.38	89	0.947	0.052	0.062	0.056–0.067

*df, degrees of freedom; CFI, comparative fit index; SRMR, standardised root mean square residual; RMSEA, root mean square error of approximation; CI, confidence interval*.

**Figure 1:**
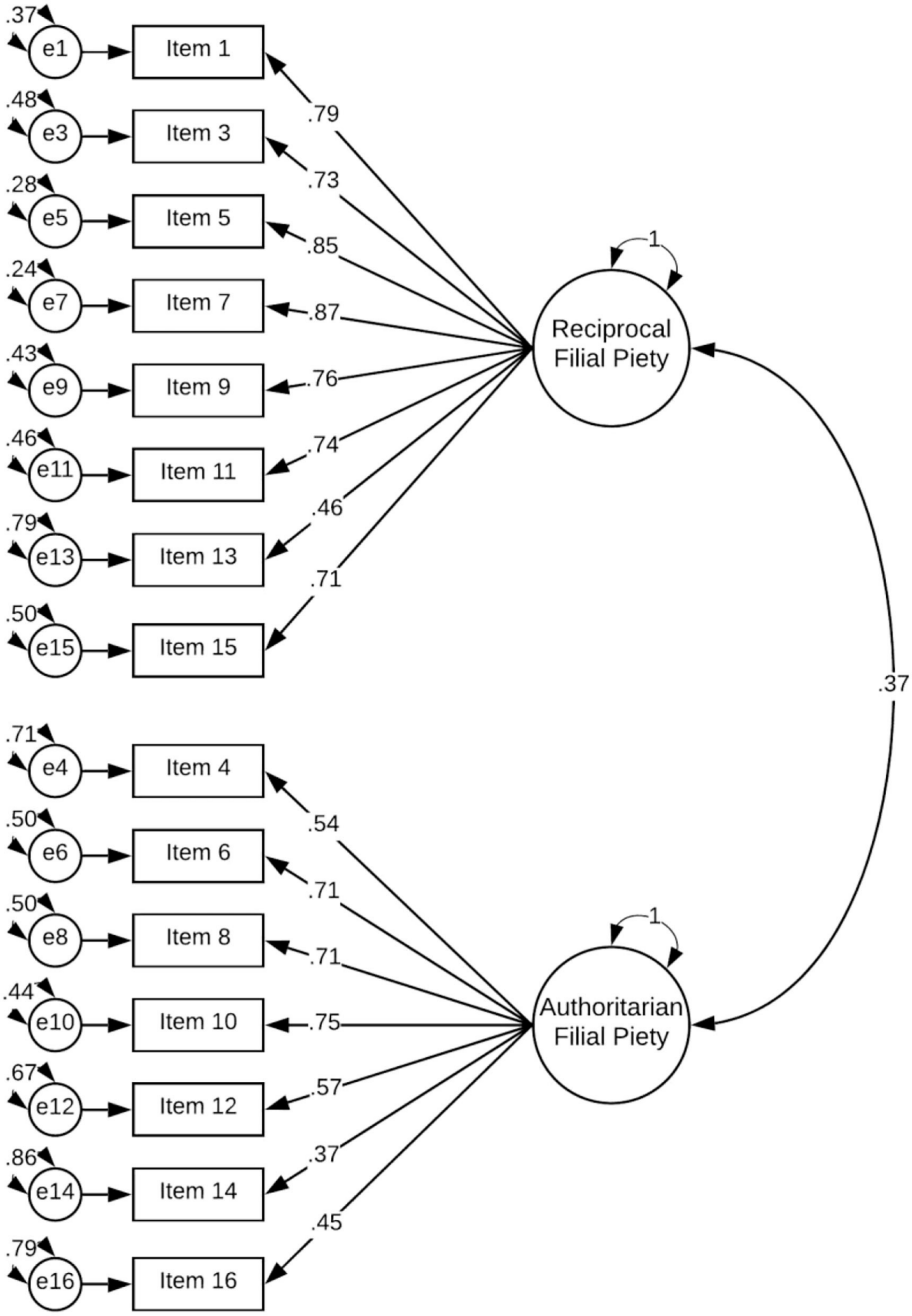
CFA results (standardised loading coefficients) of the DFPS-PL (the total sample).

When we examined the absolute fit statistics, we found that the fit of the two-factor model was acceptable (i.e., CFI > 0.90 and RMSEA < 0.08), whereas the one-factor model fit poorly. Figure 1 presents standardised loadings for the two-factor model accepted in the Polish population. The omega coefficient for the reciprocal filial piety factor was 0.93, and for the authoritarian filial piety factor, it was 0.85.

### Measurement Invariance of the DFPS-PL

Table 3 presents the global fit coefficients for the three levels of measurement invariance (configural, metric, and scalar) of the DFPS-PL across studied samples. As can be seen, the scale displayed configural and metric invariance across groups, according to the cut-off criteria suggested by Chen (2007). It showed that the students and the employees, as well as the men and the women understood the meaning of the latent construct of filial piety in the same way. However, because of lack full of scalar invariance, we tested for partial scalar invariance, releasing selected items (item 13 and item 16 form student-employee groups and item 14 and item 16 for gender groups) that varied most between the samples. Results indicated partial scalar invariance of the DFPS-PL, and thus, the means of latent variables can be compared between the groups.

**Table 3:** Global fit measures in measurement invariance tests for the DFPS-PL.

**Grouping variable**	**Level of invariance**	**χ^2^**	**df**	**CFI**	**RMSEA**	**ΔCFI**	**ΔRMSEA**
Students vs. employees	Configural invariance	590.31	178	0.942	0.065	–	–
	Metric invariance	621.61	191	0.940	0.064	0.002	0.001
	Partial scalar invariance^a^	697.61	202	0.932	0.066	0.008	0.002
	Scalar invariance	757.51	204	0.924	0.069	0.016	0.005
Men vs. women	Configural invariance	548.73	178	0.945	0.063	–	–
	Metric invariance	579.71	191	0.943	0.062	0.002	0.001
	Partial scalar invariance^b^	697.60	202	0.933	0.066	0.010	0.004
	Scalar invariance	708.00	204	0.928	0.067	0.015	0.005

*χ^2^, chi square; df, degrees of freedom; CFI, comparative fit index; RMSEA, root mean square error of approximation;*

a *intercepts for items 13 and 16 were released*;

b *and intercepts for items 14 and 16 were released*.

### Differences in Filial Piety Between the Examined Groups

To demonstrate differences in reciprocal and authoritarian filial piety among women and men in different study groups (students vs. employees), we used a two-factor ANOVA (2 × 2) scheme. We used standardised factor scores of the dependent variables to compare. As hypothesised, men (*M* = −0.28, *SD* = 0.97), regardless of whether they study or work, obtained significantly lower latent mean scores for RFP than women (*M* = 0.12, *SD* = 0.86), (*F* = 56.71, *p* < 0.01). However, we found no significant differences between the students and the employees in RFP (*F* = 0.89, *p* = 0.35). The interaction between these terms was also nonsignificant (*F* = 0.09, *p* = 0.77). Additionally, as expected, the employees (*M* = −0.03, *SD* = 0.78) obtained significantly lower latent mean scores for AFP than did the students (*M* = 0.05, *SD* = 0.68), (*F* = 3.81, *p* < 0.05). However, there were no significant differences between the men (*M* = −0.01, *SD* = 0.74) and the women (*M* = 0.01, *SD* = 0.75), (*F* = 0.12, *p* = 0.73) nor for the interaction effect (*F* = 0.02, *p* = 0.88) when AFP was compared.

## Discussion

In the given study, two main hypotheses were tested: (1) in the Polish context, filial piety attitude can be explained by two dimensions: reciprocal and authoritarian filial piety; (2) the Polish version of the DFPS demonstrates scalar measurement invariance across student-employee and gender groups; also, we investigated the student-employee and gender differences in filial beliefs in the Western culture.

The findings supported the hypothesis about two-dimensional structure of DFPS-PL and measurement invariance between the groups, which means that the scale could be used in cross-cultural comparison in future studies. However, the results revealed that not all the items of the original scale had high-factor loadings. First, one item (No. 2. *Take suggestions of my parents even when I do not agree with them*), which was removed from the final model tested in a Polish sample, loads equally both the AFP (originally assigned) and the RFP factor. In the Polish context, taking the suggestions of parents may mean listening to what the parents have to say, not necessarily acting coercively, so the Polish wording of this item may not sound strongly authoritarian. Second, there were three items (No. 13. *Hurry home upon the death of my parents, regardless of how far away I am;* No. 14. *Have at least one son for the succession of the family name; and* No. 16. *Live with my parents or parents-in-law when married*) that comprised a lot of residual variances, which may indicate that these items do not measure the constructs well in the Polish context. Item 13 in the Polish culture is associated with a very emotional situation. Considering that the studied sample consists of students and employees living in Poland, and the distances between the most distant parts of the country can be covered in a maximum of several hours, arriving at the family home due to the death of a parent is not problematic. Moreover, according to the Polish Labour Code, an employee is entitled to a special leave due to the death of the closest family members. This context explains the relatively high average score for this item. In the case of items 14 and 16, they relate to issues that may be inappropriately recognised by the respondents, especially the younger generation. Traditionally, in Poland, women take surnames of their husbands after marriage, and children are given their surnames after their fathers. Nevertheless, the law allows women to keep their maiden names or take two-part surnames, and give their children two-part surnames after both parents. Moreover, in Poland, a man may change his surname to that of his wife. Therefore, since the change of the surname is a decision of the partners, item 14 may be ambiguous for the Polish respondents. Finally, item 16 refers to living with parents after marriage. In the Polish context, this is a rarity dictated by economic considerations rather than obligations towards parents. Moving out of the family home is most often a decision supported by the parents; it indicates the independence of a child and is considered a natural course of events. Adult children live with their parents as their parents grow older and require care. Hence, once again, this item may be ambiguous for the Polish respondents.

According to other hypotheses, the lower level of AFP among employees compared to students may be related to self-reliance and the loosening of the bonds between adult children and parents. Students, who are often financially supported by their parents or are fully dependent on them, may feel grateful and have a moral obligation to take into account the views of their parents on vital issues, such as choosing a partner or a career path. For working adults in Poland, the norm seems to be fading. However, the question still remains: is it characteristic of the Western culture only? Our result, which shows that Polish women declare a higher level of RFP, suggests the gendered nature of filial piety even in the Western (i.e., Polish) culture: filial beliefs seem to be connected with defining the role of women in society as providing care for others. However, future studies are needed in this domain, especially in cross-cultural comparisons.

The present study contributes to the literature in two ways. First, the results suggest that the two-factor structure of the DFPS-PL is confirmed, and the development of the Polish DFPS adds another measure of filial beliefs for the Polish population. Also, the given study is a useful expansion of the DFPS to access filial attitudes in a new Western context (i.e., the Polish sample). Moreover, the test of its utility is understanding how transfer of filial beliefs (or do not transfer) from the role of a student to that of an employee has the potential to serve several important theoretical and practical benefits to this domain. In future studies, we would like to verify the fit of this model and elaborate on how different cultures exhibit these beliefs over time. Such a useful tool opens the door to the advancement of local as well as cross-cultural filial piety research.

Our findings suggesting the school-to-work transition and gendered nature of filial beliefs in the Western culture are consistent with Hui et al. (2018), showing that filial beliefs can be used as self-regulatory strengths, and only reciprocal (gratitude-based) filial piety supports the career ability, whereas authoritarian (submissive-based) filial piety decreases during the school-to-work transition. As filial piety is said to be the root of all virtues of social order, it deeply impacted individual attitudes and organisational behaviours (Li et al., 2021), working towards a peaceful and harmonious society (executing their social responsibilities, e.g., in business; Low and Ang, 2012).

To summarize, this paper delivers the proof that DFPS-PL is valid and could be used also in Western cultures for different social groups, i.e., both for women and men, students, and employees. What is more, the given studies suggest the school-to-work transition of filial beliefs in the society. The theoretical implication and the main conclusion are that our findings support both the *etic* nature of DFPM underlying its universal characteristic but also the *emic* character of filial piety, depending on culture (i.e., individual differences in gender or social position). Our research broadens the context for diagnosing the Eastern concept of filial piety in the Western culture by also taking ongoing social changes into account (i.e., tighter or looser cultures; Gelfand et al., 2011).

The practical implication is to provide a Polish version of a tool to measure filial piety, i.e., DFPS-PL, which can be utilised in the study of relationships and behavioural patterns in the family according to culture, where, for example, it has been proved that Chinese families attach more importance to the father-son axis rather than the husband-wife axis as compared to the Western culture (Fei, 1983). This major cultural difference may explain many communication misunderstandings within the organisation, in the employer-employee relationship, and in the relationships between men and women from different cultures brought together in a single corporation. The practical application of DFPS-PL appears on different levels and concerns different factors, such as the elder care policy due to population ageing (the social welfare system in Western countries with financial and health care support vs. child support in Asian countries), elder well-being depending on a provider of financial, housework, and health care support (i.e., the social system vs. the responsibilities of children), multicultural counselling, or academic success depending on motivational beliefs defined as filial duty, which could be analysed in cross-cultural/societal comparisons (see also: Tsao and Yeh, 2019).

## Limitations and Future Studies

In our study, some items seem to be problematic, as was already discussed; in the context of future cross-cultural comparisons, we recommend using the full 16-item scale and to monitor the problem. Partial scalar (not scalar) measurement invariance across student-employee and gender groups indicates that the employees and the students (as well as the women and the men) presenting the same level of latent variables of RFP and AFP responded differently. Fortunately, in our study, we found a problem with only two items per group—this does not significantly limit the possibility of comparing latent mean scores, but the problem should be subjected to monitoring and control in future studies.

It must be noted that the Polish population (Poland represents the Western culture) has its own specificity, which may limit the generalisation of results in the context of the child-parent relationships. Other Western cultures should be investigated, and the findings should be compared with Eastern societies. Another potential limitation is such that the differences between the employee and student groups could be due to differences in the average age between the groups, which was 17 years. The generational differences between both samples could bias the research results, showing not only the differences resulting from the stage of life (studying vs. work) but also from changing social norms regarding the relationship between children and parents over the years. Since accession of Poland to the European Union in 2004, Polish society has experienced many changes that have an impact on the system of values and attitudes (e.g., Favero, 2020), which most likely have an impact on filial beliefs.

Furthermore, given the importance of close relationships and whether people move away from home in explanation of student-employee differences in filial piety beliefs, a certain limitation of the current study is that these variables (e.g., relationship status, living with parents or not) were not collected. Nevertheless, there is, to date, little research on filial piety in the Western psychology. Our findings and the confirmed DFPS-PL scale open the door for future investigations.

## Data Availability Statement

The raw data supporting the conclusions of this article will be made available by the authors, without undue reservation. Requesting data should be addresses to Paweł Jurek, pawel.jurek@ug.edu.pl.

## Ethics Statement

The studies involving human participants were reviewed and approved by Ethics Board for Research Projects at the Institute of Psychology, University of Gdansk. Written informed consent for participation was not required for this study in accordance with the national legislation and the institutional requirements.

## Author's Note

Correspondence concerning this article should be addressed to JR-T, Institute of Psychology, University of Gdansk, Bazynskiego 4, 80-309 Gdansk, Poland; e-mail: joanna.tran@ug.edu.pl.

## Author Contributions

JR-T and PJ designed the study and wrote the paper. PJ and MO collected the data and made statistical analysis. TD gave the comments and suggestions. JR-T and TD collected funding.

## Conflict of Interest

The authors declare that the research was conducted in the absence of any commercial or financial relationships that could be construed as a potential conflict of interest.

## Publisher's Note

All claims expressed in this article are solely those of the authors and do not necessarily represent those of their affiliated organizations, or those of the publisher, the editors and the reviewers. Any product that may be evaluated in this article, or claim that may be made by its manufacturer, is not guaranteed or endorsed by the publisher.
